# Maternal Perinatal Depression, Alexithymia, and Couple Functioning: Which Relationship Exists with Prenatal Attachment

**DOI:** 10.3390/bs14090773

**Published:** 2024-09-02

**Authors:** Sonia Mangialavori, Andrea Fontana, Grazia Terrone, Eleonora Topino, Lucrezia Trani, Valeria Trincia, Giulia Lisi, Giuseppe Ducci, Marco Cacioppo

**Affiliations:** 1 Department of Pathophysiology and Transplantation, University of Milano, 20122 Milan, Italy; 2 Department of Human Science, Libera Università Maria Santissima Assunta, LUMSA, 00193 Rome, Italy; a.fontana2@lumsa.it (A.F.); e.topino@lumsa.it (E.T.); l.trani@lumsa.it (L.T.); m.cacioppo@lumsa.it (M.C.); 3 Department of History, Cultural Heritage, Education and Society, University of Rome Tor Vergata, 00133 Rome, Italy; grazia.terrone@uniroma2.it; 4 Department of Mental Health, ASL Roma 1, 00135 Rome, Italy; valeria.trincia@aslroma1.it (V.T.); giulia.lisi@aslroma1.it (G.L.); giuseppe.ducci@aslroma1.it (G.D.)

**Keywords:** maternal perinatal depression, alexithymia, dyadic adjustment, prenatal attachment

## Abstract

Background: Prenatal attachment refers to the affective investment that parents develop towards their unborn baby during pregnancy. Studies have identified depressive symptoms, affect dysregulation, and poor marital adjustment as potential risk factors for poor prenatal attachment. However, no research has concurrently examined these factors. This study aims to explore the simultaneous impact of depressive symptomatology, alexithymia, and couple functioning on prenatal attachment to develop a more comprehensive understanding of the factors shaping the emotional bond between expectant mothers and their fetuses. Methods: This cross-sectional study involved 344 women (mean age = 34.1, SD = 4.6) in their last trimester of pregnancy recruited from the National Health System. The participants completed the Edinburgh Postnatal Depression Scale, the Twenty-Item Toronto Alexithymia Scale, the Dyadic Adjustment Scale, and the Maternal Antenatal Attachment Scale. Results: Regression analyses indicated that perinatal depression and alexithymia negatively affected the Quality of Prenatal Attachment, while Dyadic Cohesion served as a protective factor. Conclusions: The results emphasize the need for early identification of perinatal depression and alexithymia, along with targeted interventions aimed at supporting Dyadic Cohesion during pregnancy. These efforts are crucial for fostering positive prenatal attachment and enhancing maternal mental health.

## 1. Introduction

The perinatal period represents a critical transitional phase for women and their families, characterized by substantial physical, physiological, and psychological changes [1,2]. This period encompasses not only biological adaptation but also significant psychological and relational shifts, requiring expectant mothers to reorganize their internal experiences and integrate new parental responsibilities, roles, and relationships with pre-existing ones [3]. Pregnancy, in particular, initiates the development of an emotional connection with the unborn child, a process grounded in Bowlby’s attachment theory [4].

In recent years, there has been a growing recognition of the importance of examining the mother–child attachment relationship from a multifaceted perspective, including not only the traditional focus on the postpartum period but also the prenatal period, as both are formative in establishing and shaping attachment bonds [5]. This broader perspective is essential because the foundations of attachment are laid during pregnancy, with maternal psychological well-being and expectations significantly influencing the early stages of the mother–child bond [6,7]. Understanding attachment through this expanded lens aligns with Bowlby’s attachment theory, which emphasizes the continuity of attachment processes across the lifespan [4].

During pregnancy, expectant mothers engage in a psychological process of adjustment that mirrors the attachment formation seen postnatally. This process begins with feelings of ambivalence and disorientation in the first trimester, as women grapple with new mental representations of themselves and their future babies [8]. As the pregnancy progresses, particularly by the second trimester, women start to accept their changing physical and psychological reality [9], a process that is deepened by the perception of fetal movements, which occurs between the end of the second and the beginning of the third trimester. These movements help mothers distinguish between themselves and the fetus, recognizing it as an entity with its own needs and intentionality [10]. This growing recognition is essential for the development of prenatal attachment, or antenatal attachment, which refers to the emotional bond that forms between a mother and her fetus during pregnancy [11,12,13]. Unlike postnatal attachment, prenatal attachment is unidirectional and abstract, focusing on the parents’ emotions, thoughts, and behaviors toward the fetus, such as naming, interacting, talking, stroking the belly, and providing prenatal care. Cranley [11] defined it as the degree of maternal behaviors representing interaction with the “unborn child”. Müller [12] emphasized the role of fantasies and thoughts, while Condon [13] highlighted the protective aspect, involving maternal tendencies to know, be with, protect, gratify needs, and avoid loss concerning the fetus.

Several maternal characteristics have been identified as influencing prenatal attachment, including affect regulation, anxiety, depression, social support, and the quality of the relationship with one’s partner [14,15,16,17,18]. These factors are crucial in shaping the emotional bond between a mother and her fetus, which is essential for the future well-being of both. Among these characteristics, alexithymia—characterized by difficulties in identifying and expressing emotions—has been found to negatively impact prenatal attachment. Alexithymic mothers may struggle to connect emotionally with their unborn child, leading to weaker prenatal attachment bonds [17]. Moreover, alexithymia is often associated with elevated levels of depression, which can further complicate the ability to form a secure attachment to the fetus [17]. This is particularly significant as numerous studies have identified an association between prenatal depressive symptoms and poor prenatal attachment [15,19,20,21,22]. These associations persist even when participants experience normal to moderate levels of depressive symptoms [20].

The influence of alexithymia and depression on prenatal attachment may be particularly pronounced in cultural contexts where emotional expressiveness and close family bonds are highly valued. For example, in a study involving Italian expectant mothers, researchers observed a negative correlation between “external-oriented thinking,” a dimension of alexithymia, and antenatal attachment, suggesting challenges in maternal imaginative and emotional investment towards the fetus [23]. In Italian culture, where expressive communication and emotionality are deeply embedded in social and familial interactions, mothers with alexithymic traits may face additional challenges in meeting societal expectations of emotional expressiveness, thereby complicating their ability to establish a strong prenatal attachment [23].

Another critical factor influencing prenatal attachment is marital adjustment [16]. The quality of the marital relationship plays a significant role not only in the overall adjustment to pregnancy but also in the bond formed with the fetus. According to attachment theory, a mother’s feelings of being loved and supported by her partner enhance her capacity to develop affection for her child [24]. This aligns with Bowlby’s concept of the secure base, where a supportive partner relationship acts as a foundation for the mother to confidently form an attachment with her unborn child [4]. Studies consistently show that emotional distance and lack of support from a partner can negatively impact parental adjustment, underscoring the importance of marital dynamics in the transition to parenthood [25,26,27]. Lower levels of marital adjustment and perceived support from partners are associated with diminished levels of prenatal attachment to the fetus [16,18,20,28]. This effect may be especially relevant in cultures like Italy, where family bonds and close-knit relationships are highly valued, and the quality of the romantic relationship can significantly influence the prenatal attachment process. A supportive and satisfying romantic relationship, characterized by closeness and mutual support, is crucial in fostering a strong emotional bond with the fetus [14]. Conversely, if a mother perceives emotional distance or lack of support from her partner, it can exacerbate feelings of isolation or stress, further impeding the development of a secure attachment to her unborn child [16].

Despite separate investigations into the roles of alexithymia, depression, and couple functioning in predicting and explaining prenatal attachment, there remains a gap in understanding how these factors interact concurrently. Previous research has explored the relationship between these variables in the postnatal period. For instance, a study evaluating mother–infant interactions in children aged 18 to 48 months found that high levels of maternal alexithymia were negatively correlated with the quality of the mother–infant relationship. Additionally, mothers with high alexithymia exhibited higher levels of depression and lower marital satisfaction [29].

This study aims to explore the simultaneous impact of these variables on prenatal attachment, thereby developing a more comprehensive understanding of the factors that shape the emotional bond between expectant mothers and their fetuses. By integrating these constructs, this study contributes to the broader attachment theory by highlighting the complexity of prenatal attachment and the multifaceted influences that shape its development. This integrated approach not only offers new insights into the early origins of the mother–child bond but also has implications for interventions aimed at promoting secure attachment from the very beginning of life.

## 2. Materials and Methods

### 2.1. Hypothesis

In line with the literature, our hypotheses were as follows:

**H1.**  Dimensions of alexithymia would be negatively associated with prenatal attachment.

**H2.**  High levels of depression would be negatively associated with prenatal attachment.

**H3.**  Dimensions of couple functioning would be positively associated with prenatal attachment.

### 2.2. Participants and Procedure

This study included 362 primiparous pregnant women in their third trimester, with an average age of 33.69 years (SD = 4.61; range 22–48 years).

Participants were recruited from the ASL Rome 1 Mental Health District of the National Health System (NHS) in the metropolitan city of Rome, Italy. Due to COVID-19 restrictions, recruitment took place through online prenatal support courses throughout 2022. Although the Italian government ended the state of emergency for COVID-19 on 31 March 2022, these prenatal support courses continued to be conducted online throughout the year.

Data collection occurred via an online platform and participants were informed about the research objectives before starting the survey. They accessed the platform through an anonymous link and were required to review and sign an informed consent form. Both verbal and written consent were obtained before enrollment, ensuring that participation was voluntary, and privacy was maintained.

Inclusion criteria required participants to be in their third trimester of pregnancy, primiparous, fluent in Italian, and at least 18 years old. Exclusion criteria were having a high-risk pregnancy defined as the presence of one or more maternal and/or fetal health problems including pregnancy-induced hypertension, multiple gestations, medical disorder complicating pregnancy (such as diabetes), previous miscarriages, chromosomal abnormalities in the fetus, pregnancy complications (such as abnormal placenta position, fetal growth restriction) and threatened premature labor, refusal to provide informed consent, cognitive disabilities or psychiatric diagnoses, insufficient proficiency in Italian, or other communication barriers that could hinder adherence to the research protocol.

All participants identified as White Europeans.

As presented in Table 1, the majority of participants were either cohabiting (48%) or married (41%), with a smaller percentage identifying as single (6%) or separated (1%). Additionally, a noteworthy proportion of participants were employed (80%), and a majority reported possessing a university degree (71%). Confidentiality of information was meticulously maintained, with data presented in an aggregated format. This study adhered to the Ethical Code of the American Psychological Association (APA), the Declaration of Helsinki, and the Ethical Guidelines of the Italian Psychological Association (AIP).

### 2.3. Measures

#### 2.3.1. Maternal Antenatal Attachment Scale (MAAS)

The Maternal Antenatal Attachment Scale (MAAS) [13,30] was used to assess the Quality of Prenatal Attachment. The MAAS is a self-report measure consisting of 19 items, scored on a 5-point Likert scale (from 1 to 5). Both a total score and two subscales can be calculated: (1) quality of attachment, which evaluates the quality of the maternal emotional bond with the fetus, and (2) intensity of preoccupation, which evaluates the frequency and intensity of preoccupation with the fetus. Higher scores indicate a higher quality of attachment. The Italian version of the quality of attachment subscale was used in the present research and showed satisfactory internal consistency in the present sample (ω = 0.69).

#### 2.3.2. Twenty-Item Toronto Alexithymia Scale (TAS-20)

The Twenty-Item Toronto Alexithymia Scale (TAS-20) [31,32] was used to assess the levels of alexithymia. The TAS-20 is a self-report measure consisting of 20 items scored on a 5-point Likert scale (from 1 to 5). Both a total score and three subscales (difficulty identifying feelings; difficulty describing feelings; and externally oriented thinking) can be calculated. Higher scores indicate higher levels of alexithymia. The Italian version was used in this research and showed satisfactory internal consistency in the present sample (total score, ω = 0.81; difficulty identifying feelings, ω = 0.80; difficulty describing feelings, ω = 0.71; and externally oriented thinking, ω = 0.61).

#### 2.3.3. Edinburgh Postnatal Depression Scale (EPDS)

The Edinburgh Postnatal Depression Scale (EPDS) [33,34] was used to assess the levels of perinatal depression. The EPDS is a self-report measure consisting of 10 items, scored on a 4-point Likert scale (from 0 to 3). A higher score indicates higher levels of perinatal depression symptomatology. In greater detail, a cut-off score of ≥9 was indicative of moderate symptomatology and the need for initial support, whereas a cut-off score of ≥13 was employed to identify individuals with a higher likelihood of experiencing depression [34,35]. The Italian version demonstrated excellent sensitivity in evaluating depression in both pre- and postnatal periods [30] and showed good internal consistency in the present sample (ω = 0.79).

#### 2.3.4. Dyadic Adjustment Scale (DAS)

The Dyadic Adjustment Scale (DAS) [36,37] was used to assess the levels of Dyadic Adjustment. The DAS consists of 32 items rated with different response formats and grouped into four subscales: Dyadic Consensus (the degree to which the couple agrees on matters of importance to the relationship), Dyadic Satisfaction (the degree to which the couple is satisfied with their relationship), Dyadic Cohesion (the degree of closeness and shared activities experienced by the couple), and Affective Expression (the degree of demonstrations of affection and sexual relationships). Higher scores indicate higher levels of Dyadic Adjustment. The Italian version was used in this research and showed satisfactory internal consistency in the present sample (Dyadic Consensus, ω = 0.85; Dyadic Satisfaction, ω = 0.74; Dyadic Cohesion, ω = 0.54; and Affective Expression, ω = 0.66).

### 2.4. Analytic Strategy

Statistical analyses were conducted using IBM SPSS Statistics for Macintosh, Version 27.0, and Jamovi v.2.3.19. Initially, thorough scrutiny of the dataset was undertaken to ensure data entry accuracy and identify any missing values. Little’s MCAR test was subsequently employed to assess the null hypothesis regarding the complete randomness of missing scale scores (i.e., MCAR assumption) [38]. To address missing values, we chose listwise deletion from the dataset. Univariate outliers were identified by examining z scores and box plots, while multivariate outliers were detected using the Mahalanobis distance and subsequent χ^2^ test with a significance level set at *p* < 0.001. Following this, we evaluated the normality of variable distributions by assessing skewness and kurtosis. Assumptions for multiple hierarchical regression were verified, focusing on residual independence, linearity of variable relationships, homoscedasticity, multicollinearity, and error distribution. Initially, descriptive statistics were computed for the study variables. Following this, bivariate correlations were conducted to examine the relationships between the Quality of Prenatal Attachment, alexithymia, perinatal depression, and couple functioning. Subsequently, a multiple hierarchical regression analysis was performed, with Quality of Prenatal Attachment as the outcome variable. In Step 1, difficulty identifying feelings was introduced as a predictor. Then, in Step 2, difficulty describing feelings was added to assess its association with the Quality of Prenatal Attachment beyond difficulty in identifying feelings. Step 3 involved the addition of Externally oriented thinking to explore its relationship with the Quality of Prenatal Attachment over and above the previously introduced predictors. In Step 4, perinatal depression was included to examine its relationship with the Quality of Prenatal Attachment over and above the other predictors. Finally, the four Dyadic Adjustment dimensions were sequentially added from Step 5 to Step 8 to test their relationship with the Quality of Prenatal Attachment over and above the other predictors. Standardized regression coefficients (β) were estimated, with β values of −0.1, 0.1, −0.3, 0.3, −0.5, and 0.5 or greater interpreted as representing small, medium, and large effect sizes, respectively.

An a priori power analysis was conducted using G*Power version 3.1.9.7 [39] to test a multiple regression using a medium effect size (f^2^ = 0.15), an alpha of 0.05, and eight main predictors. The results showed that a total sample of 109 participants was needed to achieve a power of 0.80. 

## 3. Results

As shown in Table 1, based on the EPDS cut-off scores, 26% of the pregnant women involved in the research reported perinatal depression levels suggesting the need for support (ranging from moderate to intense symptomatology). Meanwhile, 74% exhibited a low or no level of perinatal depression. In each scale, the prevalence of missing values was 2%, and the Little MCAR test yielded a non-significant result (χ^2^ = 15.407, DF = 13, Sig. = 0.283), suggesting a completely random pattern in the missing data. Consequently, subjects with missing values were excluded from the analysis. Subsequently, we identified nine pregnant women (2.5%) exhibiting exceptionally high z scores on the Quality of Prenatal Attachment, TAS Scales, and Dyadic Adjustment, categorizing them as univariate outliers, and they were consequently excluded from further analysis. Additionally, through the Mahalanobis distance, two more cases (0.6%) were identified as multivariate outliers with *p* < 0.001 and were subsequently excluded from the dataset. Thus, eleven outliers were excluded from the analyses: the final sample comprised 344 primiparous expectant mothers in the third trimester of pregnancy. Descriptive statistics of the investigated variables are presented in Table 2.

We ensured that the assumptions were satisfied before initiating the linear regression analysis. Linearity was confirmed through partial regression plots and a plot of Studentized residuals against the predicted values. The independence of residuals, indicated by a Durbin–Watson statistic of 1.90, was upheld. Homoscedasticity was verified through visual inspection of a plot depicting Studentized residuals against unstandardized predicted values. No evidence of multicollinearity was found, as indicated by tolerance values exceeding 0.1. Moreover, the assumption of normality was validated through a Q-Q Plot and an assessment of skewness and kurtosis for the considered variables.

Pearson correlations are detailed in Table 3, revealing a negative correlation between the Quality of Prenatal Attachment and alexithymia. Additionally, a positive correlation was observed between the Quality of Prenatal Attachment and Dyadic Adjustment. Consistent with expectations, perinatal depression exhibited a negative correlation with the Quality of Prenatal Attachment. The results of the hierarchical regression model for Quality Prenatal Attachment are in Table 4. Step 1 showed that difficulty identifying feelings significantly predicted Quality of Prenatal Attachment in pregnant women, R^2^ = 0.15, F(1, 342) = 62.2, *p* < 0.001. In Step 2, the inclusion of difficulty describing feelings yielded a significant prediction of the Quality of Prenatal Attachment, contributing to an increase in ΔR^2^ of 0.02. However, it is noteworthy that in Step 3, upon introducing externally oriented thinking into the model, it emerged as a predictor of Quality of Prenatal Attachment, concurrently suppressing the predictive role of difficulty describing feelings. The overall model in Step 3 was statistically significant, with an R^2^ of 0.21 (ΔR^2^ = 0.03) and an F(3, 340) value of 29.2, *p* < 0.001. In Step 4, perinatal depression was added to the model leading to a significant increase in R^2^ (R^2^ = 0.24; ΔR^2^ = 0.03) with an F(4, 339) value of 26.2, *p* < 0.001.

The results underscored that elevated difficulty in identifying feelings and externally oriented thinking were associated with poorer Quality of Prenatal Attachment in pregnant women. Furthermore, increased levels of perinatal depression were linked to diminished Quality of Prenatal Attachment. From Step 5 to Step 8, various dimensions of the couple’s functioning were introduced into the model. As depicted in Table 4, only Dyadic Cohesion emerged as a significant and positive contributor to the variance in Quality of Prenatal Attachment (R^2^ = 0.25; ΔR^2^ = 0.03), with an F(6, 337) value of 20.1, *p* < 0.001. This result indicates that higher Dyadic Cohesion perceived by pregnant women toward their partners corresponds to an increased Quality of Prenatal Attachment.

## 4. Discussion

This study aimed to examine the simultaneous impact of maternal perinatal depression, alexithymia, and couple functioning on the quality of prenatal attachment among first-time expectant mothers. The findings of this study provide critical insights into how these factors interact to influence prenatal bonding. Consistent with our first hypothesis, the results revealed that alexithymia significantly negatively affects prenatal attachment. Specifically, the dimensions of difficulty identifying feelings and externally oriented thinking were notable predictors. These findings are in line with previous studies suggesting that alexithymic traits hinder emotional bonding and imaginative engagement with the fetus [17,23]. The inability to recognize and articulate emotions likely disrupts a mother’s capacity to form a mental representation of the fetus and to sense affective feelings crucial for prenatal attachment. Externally oriented thinking, which involves a focus on external realities rather than internal emotional states, was a significant predictor of lower prenatal attachment quality. Indeed, this dimension refers to a kind of concrete reasoning often related to barren and frozen mental functioning, which is considered a predictor of an extremely poor mother–child relationship [23]. In line with previous research [40], this result is consistent with the association between emotion dysregulation and worse quality of prenatal attachment. This emphasizes the need for psychological interventions that enhance emotional awareness and mentalization in expectant mothers to support prenatal bonding. Moreover, these findings underscore the importance of considering alexithymia in prenatal care. Screening for alexithymic traits during routine prenatal visits could help identify mothers at risk of developing poor prenatal attachment. Interventions such as emotion-focused therapy and mentalization-based treatment, which aim to improve emotional awareness and expression, could be beneficial in enhancing prenatal attachment among mothers with high levels of alexithymia.

Supporting our second hypothesis, this study found a significant negative association between perinatal depression and the quality of prenatal attachment. This aligns with the existing literature linking higher levels of depressive symptoms during pregnancy with poorer prenatal attachment [19,21]. Depression during pregnancy can lead to a range of symptoms, including anhedonia, low energy, and pervasive negative affect, which can impede a mother’s ability to invest emotionally in her fetus. However, as noted by several authors, while depression may inhibit the development of a positive bond, it does not necessarily reduce the overall intensity of attachment; for example, the parents may still feel a strong connection but experience predominantly negative thoughts and emotions toward the fetus [20,41]. Moreover, these negative emotions and concerns toward the fetus could be exacerbated by the pandemic. Although the Italian government officially ended the state of emergency for COVID-19 on 31 March 2022, emotional residuals and fears related to the pandemic could still impact expectant mothers [42,43]. The lingering effects of pandemic-related stressors, such as heightened anxiety about health and safety, social isolation, and general uncertainty, might have intensified feelings of fear and helplessness, further complicating the emotional connection between the mother and the fetus [44,45].

Given these considerations, it is important for prenatal care providers to recognize both the direct effects of perinatal depression and the potential residual emotional impacts of the pandemic. Incorporating comprehensive mental health support and addressing pandemic-related stressors, even in a post-emergency context, is crucial for promoting positive prenatal attachment and overall maternal well-being. The findings also highlight the necessity of early screening and treatment of depressive symptoms during pregnancy to foster healthy prenatal attachment.

In this regard, the results suggest that prenatal care providers should integrate routine mental health screenings into their practice to identify depressive symptoms early. Providing access to mental health services and support groups can help manage depressive symptoms and promote a positive prenatal attachment. Various psychological interventions have shown efficacy in treating perinatal depression and could be recommended as part of a comprehensive prenatal care plan [46]. Women identified as "at risk" during early pregnancy should receive continuous support throughout the prenatal and postpartum periods to foster a secure mother–child bond. For instance, maternal–fetal attachment can be modified by specific supportive interventions that are effective in enhancing the quality of parental bonding [6,47]. These programs for parents-to-be should encourage fantasies about their baby, closeness to the future child, and care for the fetus [48,49]. As Abasi et al. [50] suggest, effective interventions should focus on enriching prenatal attachment behaviors, such as counting fetal movements, touching the belly, and talking to the fetus.

In line with our third hypothesis, aspects of couple functioning, particularly dyadic cohesion, were found to positively influence prenatal attachment quality. This finding is consistent with previous research highlighting the critical role of a supportive and satisfying romantic relationship in fostering prenatal attachment [16,20]. Dyadic cohesion, characterized by emotional and physical closeness and shared activities, likely provides a secure and emotionally supportive environment for the mother, giving room to her capacity to form a strong emotional bond with her unborn child. Interestingly, other dimensions of couple functioning, such as dyadic consensus and satisfaction, did not emerge as significant predictors, suggesting that the closeness and shared experiences inherent in dyadic cohesion are particularly vital during pregnancy. These findings underscore the importance of couple-based interventions during pregnancy. Relationship counseling and couples’ psychological interventions focused on enhancing communication, emotional closeness, and shared activities could strengthen dyadic cohesion and, in turn, improve prenatal attachment. For instance, Berthelot et al. [51] found that a brief intervention aimed at improving the psychological well-being of pregnant women led to better couple functioning, reflected in an increased interest in the partner’s psychological state. This demonstrates the positive impact of mentalization-based interventions on key attachment relationships. By enhancing prenatal couple dynamics, such interventions not only strengthen the emotional connection between partners but also facilitate the development of a positive prenatal attachment. A secure and supportive relationship between partners during pregnancy fosters a nurturing environment, which is essential for the mother to form a deep and secure bond with her unborn child. This, in turn, lays a strong foundation for effective co-parenting, emotional stability at home, and a reflective, supportive environment that benefits the child’s development from the earliest stages [7]. Furthermore, prenatal classes that include sessions on relationship enhancement and dyadic coping strategies for the changes brought by pregnancy could also be beneficial [49].

The findings of this study have significant implications for clinical practice. First, the identification of alexithymia and depressive symptoms as key predictors of prenatal attachment underscores the importance of routine psychological assessments during prenatal care. Health professionals should consider implementing screening tools for alexithymia and depression as part of standard prenatal care. Interventions focusing on enhancing emotional awareness and managing depressive symptoms could be crucial in supporting prenatal bonding. Second, the protective role of dyadic cohesion emphasizes the value of couple-based interventions during pregnancy. Strengthening the emotional bond between partners can create a nurturing environment that promotes positive prenatal attachment. Integrating these findings into clinical practice requires a multi-faceted approach. Healthcare providers should be trained to recognize signs of alexithymia and depression and refer patients to appropriate mental health services. Additionally, prenatal programs should include components that address relationship dynamics, offering resources and support to expectant couples to enhance their emotional connection and preparedness for parenthood.

Despite the strengths of this study, including its comprehensive examination of multiple predictors of prenatal attachment, several limitations must be acknowledged. The cross-sectional design limits our ability to determine causal relationships; thus, future research should employ longitudinal designs to explore how these variables interact over time and their impact on postnatal child attachment.

Additionally, this study did not account for certain obstetric variables, such as gestational age, unplanned pregnancy, or conception through in vitro fertilization (IVF), nor did it consider factors like mental health history or perceived social support, all of which could potentially influence prenatal bonding. The sample was also predominantly highly educated or had a college degree, and relatively homogeneous in terms of ethnicity and socioeconomic status, which may affect the generalizability of the findings. Additionally, women did not have at-risk pregnancies. Future research should aim to include more diverse populations, including women with high-risk pregnancies, to enhance the external validity of the results.

Furthermore, examining potential moderating and mediating factors, such as maternal age, the quality of the mother’s attachment relationships, or maternal personality traits, could provide deeper insights into the dynamics of prenatal attachment.

Future research should also investigate the potential role of paternal dyadic factors in prenatal attachment. Understanding how paternal mental health and couple dynamics influence maternal–fetal attachment could provide a more holistic view of the factors that contribute to prenatal bonding. Additionally, exploring the effectiveness of specific interventions aimed at improving alexithymia, managing depressive symptoms, and enhancing couple cohesion could inform best practices for supporting expectant mothers.

## 5. Conclusions

In conclusion, this study highlights the complex interplay between maternal psychological factors and relational dynamics in shaping prenatal attachment. The detrimental effects of alexithymia and perinatal depression, coupled with the protective influence of dyadic cohesion, underscore the need for integrated approaches in prenatal care that address both individual psychological needs and couple dynamics. Enhancing our understanding of these predictors can lead to more effective interventions aimed at fostering healthy prenatal attachment and, consequently, better outcomes for both mother and child. Specifically, interventions that strengthen dyadic cohesion and enhance maternal reflective functioning and emotional regulation could help expectant mothers to better connect emotionally with their unborn child, positively influencing their mental health. Routine prenatal care assessments should include systematic screenings for alexithymia, perinatal depression, and the quality of the couple’s relationship. Early identification of at-risk mothers would allow for timely interventions, improving both maternal and child health outcomes. Healthcare providers should therefore be trained to assess and support both couple dynamics and maternal psychological health.

Further research should also explore the long-term effects of couple dynamics and maternal psychological factors on both maternal mental health and child development beyond the prenatal period. Additionally, longitudinal studies could provide deeper insights into how pandemic-related stressors impact these variables over time. Such studies would clarify how interventions designed to enhance dyadic coping, emotional regulation, and reflective functioning may influence not only prenatal attachment but also postnatal attachment and overall child development.

Moreover, expanding research to include diverse populations in terms of age, socioeconomic status, and cultural background would enrich our understanding of these variables, making the findings more generalizable and applicable across different contexts. These insights could guide public health policies toward more inclusive and targeted interventions that support the psychological and relational well-being of expectant mothers.

## Figures and Tables

**Table 1:** Demographic characteristics of the 362 pregnant women involved in this study.

Characteristics	M ± SD	N (*%*)
Age	33.69 ± 4.61	
Marital status		
*Single*		22 (6.1%)
*Married*		149 (41.2%)
*Cohabiting*		175 (48.3%)
*Separated*		2 (0.6%)
*Missing*		14 (3.9%)
Education		
*Middle School diploma*		1 (0.3%)
*High School diploma*		88 (24.3%)
*University degree*		259 (71.5%)
*Missing*		14 (3.9%)
Occupation		
*Student*		2 (0.6%)
*Artisan*		2 (0.6%)
*Employee*		289 (79.8%)
*Freelance*		41 (11.3%)
*Trader*		12 (3.3%)
*Homemaker*		4 (1.1%)
*Unemployed*		12 (3.3%)
Symptoms of perinatal depression		
*Low or no level*		269 (74.3%)
*Moderate level*		63 (17.4%)
*Probable depression*		30 (8.3%)

**Table 2:** Descriptive statistics among the study variables.

						Skewness		Kurtosis	
	Mean	Median	SD	Minimum	Maximum	Skewness	SE	Kurtosis	SE
Prenatal attachment	46.57	47	3.84	33	53	−0.604	0.131	0.0386	0.262
TAS_F1	10.29	9	4.04	7	26	1.486	0.130	1.6742	0.260
TAS_F2	9.22	8	3.72	5	22	1.008	0.130	0.6031	0.260
TAS_F3	15.43	15	4.17	8	29	0.392	0.130	−0.3297	0.260
Perinatal depression	6.09	6	4.02	0	21	0.755	0.130	0.4052	0.260
Dyadic_Cons	55.95	56.5	5.81	35	65	−0.703	0.130	0.247	0.260
Dyadic_Satisf	44.26	45	3.83	29	51	−1.148	0.130	1.8496	0.260
Dyadic_Aff_Expr	10.35	11	1.73	4	12	−1.029	0.130	0.4187	0.260
Dyadic_Cohes	17.84	18	3.42	7	24	−0.458	0.130	−0.0573	0.260

Note: Prenatal attachment: total score of the Maternal Antenatal Attachment Scale (MAAS); TAS_F1: difficulty identifying feelings (TAS-20); TAS_F2: difficulty describing feelings (TAS-20); TAS_F3: externally oriented thinking (TAS-20); perinatal depression: total score of the Edinburgh Postnatal Depression Scale (EPDS); Dyadic_Cons: Dyadic Consensus (DAS); Dyadic_Satisf: Dyadic Satisfaction (DAS); Dyadic_Aff_Expr: Affective Expression (DAS); and Dyadic_Cohes: Dyadic Cohesion (DAS).

**Table 3:** Zero-order correlations among the study variables.

	1	2	3	4	5	6	7	8	9
1. Prenatal attachment	—								
2. TAS_F1	−0.392 ***	—							
3. TAS_F2	−0.325 ***	0.536 ***	—						
4. TAS_F3	−0.311 ***	0.265 ***	0.387 ***	—					
5. Dyadic_Satisf	0.213 ***	−0.323 ***	−0.285 ***	−0.081	—				
6. Dyadic_Aff_Expr	0.162 **	−0.317 ***	−0.282 ***	−0.087	0.557 ***	—			
7. Dyadic_Cohes	0.259 ***	−0.137 *	−0.208 ***	−0.181 ***	0.444 ***	0.344 ***	—		
8. Dyadic_Cons	0.261 ***	−0.385 ***	−0.351 ***	−0.115 *	0.631 ***	0.516 ***	0.406 ***	—	
9. Perinatal depression	−0.374 ***	0.477 ***	0.384 ***	0.253 ***	−0.327 ***	−0.285 ***	−0.159 **	−0.416 ***	—

Note: * *p* < 0.05, ** *p* < 0.01, and *** *p* < 0.001. Prenatal attachment: total score of the Maternal Antenatal Attachment Scale (MAAS); TAS_F1: difficulty identifying feelings (TAS-20); TAS_F2: difficulty describing feelings (TAS-20); TAS_F3: externally oriented thinking (TAS-20); Dyadic_Satisf: Dyadic Satisfaction (DAS); Dyadic_Aff_Expr: Affective Expression (DAS); Dyadic_Cohes: Dyadic Cohesion (DAS); Dyadic_Cons: Dyadic Consensus (DAS); and perinatal depression: total score of the Edinburgh Postnatal Depression Scale (EPDS).

**Table 4:** Summary of hierarchical regression analysis for the association between alexithymia, perinatal depression, and Couple Dyadic Adjustment with Quality of Prenatal Attachment.

Predictor	B	*SE*	t	*p*	β	Lower	Upper
Intercept	49.611	2.800	17.717	<0.001			
TAS_F1	−0.209	0.058	−3.629	<0.001	−0.220	−0.3396	−0.1008
TAS_F2	−0.050	0.061	−0.812	0.417	−0.048	−0.1657	0.0689
TAS_F3	−0.143	0.048	−2.979	0.003	−0.155	−0.2572	−0.0526
Perinatal depression	−0.186	0.054	−3.441	<0.001	−0.194	−0.3057	−0.0833
Dyadic_Aff_Expr	−0.124	0.131	−0.952	0.342	−0.056	−0.1713	0.0596
Dyadic_Cohes	0.198	0.061	3.245	0.001	0.175	0.0689	0.2811
Dyadic_Satisf	−0.002	0.067	−0.030	0.976	−0.002	−0.1306	0.1267
Dyadic_Cons	0.013	0.044	0.298	0.766	0.020	−0.1097	0.1488

Note: TAS_F1: difficulty identifying feelings (TAS-20); TAS_F2: difficulty describing feelings (TAS-20); TAS_F3: externally oriented thinking (TAS-20); perinatal depression: total score of the Edinburgh Postnatal Depression Scale (EPDS); Dyadic_Aff_Expr: Affective Expression (DAS); Dyadic_Cohes: Dyadic Cohesion (DAS); Dyadic_Satisf: Dyadic Satisfaction (DAS); and Dyadic_Cons: Dyadic Consensus (DAS).

## Data Availability

The data presented in this study can be obtained from the corresponding author upon reasonable request, as they are not publicly available due to further ongoing analysis.
